# Prostatic Abscess on Xanthogranulomatous Prostatitis: Uncommon Complication of an Uncommon Disease

**DOI:** 10.1155/2018/5417903

**Published:** 2018-08-29

**Authors:** Youness Jabbour, Hamza Lamchahab, Sumba Harrison, Hafsa El Ouazzani, Tarik Karmouni, Khalid El Khader, Abdellatif Koutani, Ahmed Iben Attya Andaloussi

**Affiliations:** ^1^Urology B Department, Ibn Sina Teaching Hospital, Rabat, Morocco; ^2^Faculty of Medicine and Pharmacy, Mohammed V University, Rabat, Morocco; ^3^Anatomical Pathology Department, Ibn Sina Teaching Hospital, Rabat, Morocco

## Abstract

Xanthogranulomatous prostatitis is a rare benign inflammatory process of the prostate. Only few cases have been reported in the English literature. Xanthogranulomatous prostatitis is usually an incidental finding after needle biopsy or transurethral resection of the prostate in patients suffering from low urinary tract symptoms. We report the case of a 59-years-old patient diagnosed with prostatic abscess managed by transurethral resection of the prostate. Histopathological examination of resected prostatic tissue revealed abscessed xanthogranulomatous prostatitis with no evidence of malignancy. Xanthogranulomatous prostatitis presenting as a prostatic abscess is a rare finding. To the best of our knowledge our case represents the fourth case of xanthogranulomatous prostatitis presenting as prostatic abscess reported in the English literature so far.

## 1. Introduction

Granulomatous prostatitis is an unusual inflammatory process of the prostate gland characterized by the presence of granuloma as the main histological feature. It was first described by Tanner and McDonald in 1943 and [1, 2].

A variety of granulomatous lesions of the prostate have been described with varied etiology and pathogenesis leading to their classification by Epstein and Hutch into various types including nonspecific (idiopathic), specific (infectious), iatrogenic (postsurgery, postradiation), malakoplakia, and associated systemic granulomatous disease. The nonspecific type of granulomatous inflammation is the most common [2, 3].

This classification although being controversial remains widely used.

Xanthogranulomatous prostatitis is a rare form of nonspecific granulomatous prostatitis distinguished by presence of xanthoma cells.

We report an additional case of xanthogranulomatous prostatitis revealed by prostatic abscess.

## 2. Case Presentation

We report the case of a 59-years-old diabetic patient, without particular medical history, experiencing urgency, frequency, hesitancy, intermittency, straining, and slow stream and for three weeks.

**Figure 1:**
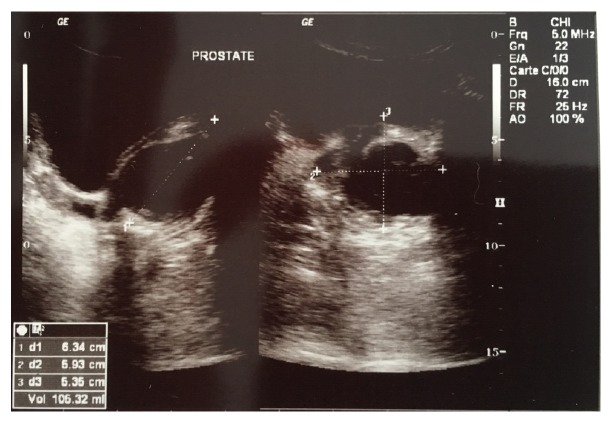
Abdominal ultrasound showing hypoechoic collection inside prostatic parenchyma.

He presented a recent onset of fever, myalgia, chill, and an episode of gross hematuria for which he was seen in a local hospital. Abdominopelvic ultrasonography showed thickness of the bladder wall without associated process and a hypoechogenic and enlarged prostate measuring 106,32 ml. Postmicturition residual urine was 143 ml (Figure 1).

Laboratory analysis showed no coagulation abnormalities with a platelet count within normal limits, high erythrocyte sedimentation rate of 94 mm/h, and high white cell count of 23880 cells/mm^3^ with 90,6% of neutrophils.

Urine analysis revealed hematuria and leukocyturia with no growth in urine culture. His renal function tests were normal.

**Figure 2:**
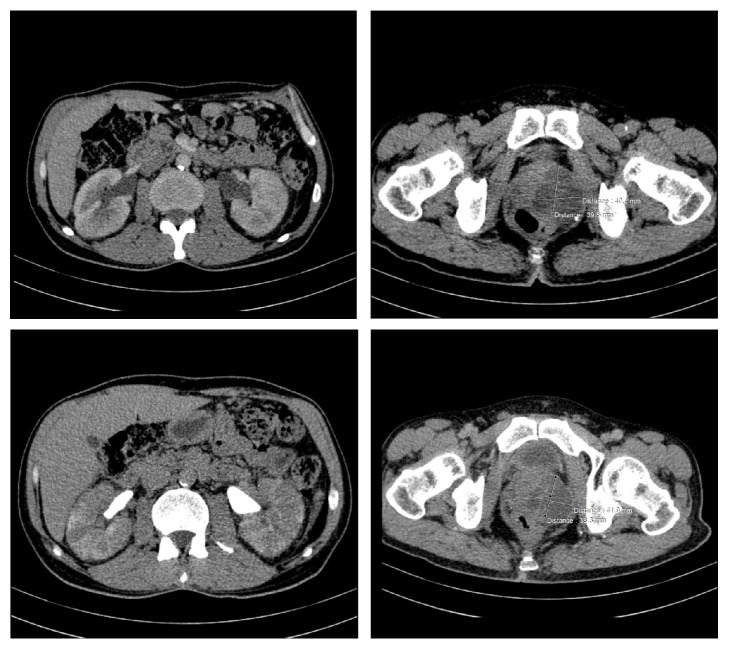
Tomodensitometric views showing prostatic abscess with bilateral ureterohydronephrosis.

A subsequent computed tomography urography was performed revealing a prostatic abscess measuring 50 mm in diameter enlarging the prostate with infiltration of the periprostatic fat. The bladder was distended without anomalies within its wall. Bilateral ureterohydronephrosis with normal renal parenchyma was also noted (Figure 2).

Urethral catheterization was performed and he was addressed to urology department.

Physical exam at the time of presentation to urology department found a patient in good general health apyretic. His vital signs were within normal limits. Digital rectal examination was painful and found a firm and enlarged prostate without nodules. Transrectal ultrasound was not tolerated by the patient. His International Prostate Symptom Score (IPSS) was 26 consistent with severe low urinary tract symptoms (LUST). We did not perform a prostate-specific antigen (PSA) test since patient provided us with a former test done one month earlier to his admission which was at 0,54 ng/ml. Also, since he has a prostatic abscess PSA was expected to be elevated.

**Figure 3:**
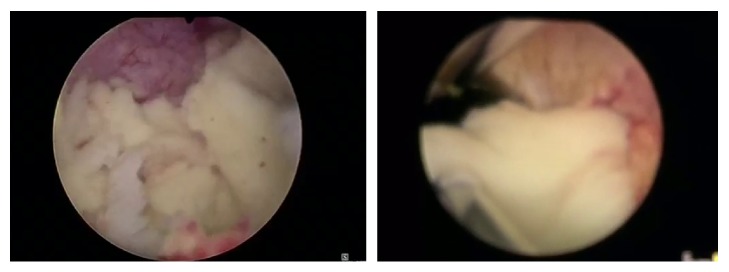
Endoscopic view showing important amounts of thick whitish pus.

The patient underwent a transurethral resection of the prostate (TURP) showing an important enlargement of the left lateral lobe obstructing the prostatic urethra. Resection permitted an unroofing of the abscess and a drainage of abundant amounts of thick whitish pus and allowed collapse of pus pockets under control of view (Figure 3).

**Figure 4:**
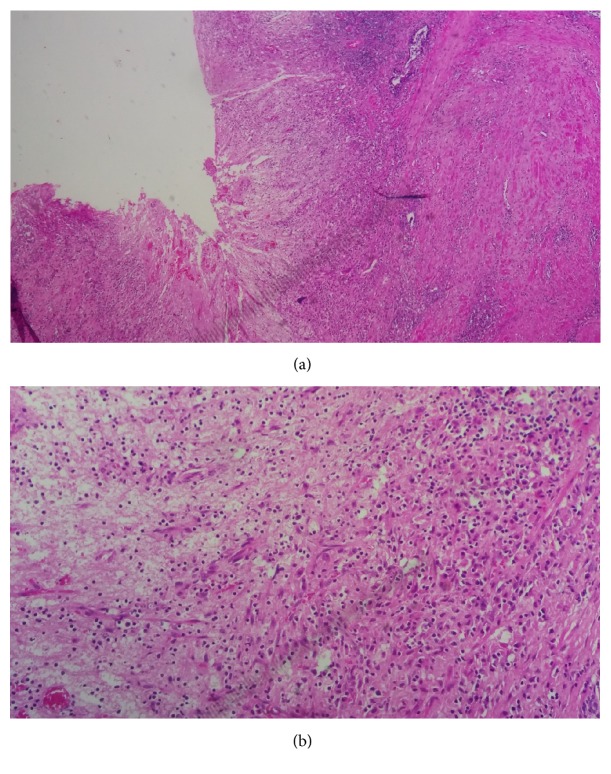
Microscopic view of resected specimen. (a) Prostatic parenchyma seat of an abscessed collection bordered by a polymorph inflammatory infiltrate. (b) Xanthomatous infiltrate rich in foamy histiocytes without presence of gigantocellular or epithelioid granuloma.

His postoperative recovery was uneventful. Tissue cultures were negative. Histologic examination of resected chips concluded on suppurated xanthogranulomatous prostatitis (Figure 4).

## 3. Discussion

Xanthogranulomatous inflammation is well known in the kidney and gallbladder and has been described in many anatomic sites, such as the mandible, retroperitoneum, third ventricle, choroid plexus, orbit, vagina, lung, stomach, pericardium, and ovary [4].

Since its first description only few cases of xanthogranulomatous prostatitis not exceeding 20 have been reported in the English literature [5].

The exact etiology of xanthogranulomatous prostatitis is still uncertain. Several theories tending to explain its pathogenesis have been advocated (hyperlipidemia, autoimmunity, ductal obstruction,…) [4, 6, 7].

Among them ductal obstruction appears to play a major role in its pathogenesis.

Bostwick and Chang advanced that after blockage of prostate ducts and stasis of secretions, the cellular debris, bacterial toxins, prostatic secretions, including corpora amylacea, sperm, and semen escape into the stroma through the destroyed epithelium, eliciting an intense localized inflammatory response [4].

Xanthogranulomatous prostatitis is mostly observed in adult patients with an average age at the time of diagnosis being early sixties [8–10].

The most frequent and encountered clinical presentations in former reported cases of xanthogranulomatous prostatitis were lower tract urinary symptoms, digital rectal examination anomalies, and transient elevation in serum PSA level that reached 172 ng/ml in two patients [4, 11].

Age of occurrence, hard and nodular prostate on digital rectal examination, and raised serum PSA levels make xanthogranulomatous prostatitis a difficult differential diagnosis of prostatic adenocarcinoma.

Additionally, no imaging study (rectal ultrasound, MRI) could differentiate with confidence between xanthogranulomatous prostatitis and prostatic adenocarcinoma given radiology resemblances as reported by Cheng et al. [7]

Final diagnosis of xanthogranulomatous prostatitis is exclusively based on histopathological examination.

Pathologically xanthogranulomatous prostatitis shows features of lobulocentric accumulation of inflammatory cells including lymphocytes, plasma cells, and sometimes polymorphs with eosinophils [7, 8].

The distinctive feature of xanthogranulomatous prostatitis is the presence of large number of foamy histiocytes (lipid-laden macrophages) also known as xanthomatous cells admixed with other inflammatory cells. Immunohistochemistry reveals T lymphocytes in close association with damaged epithelium while B lymphocytes occur in more peripheral location or form follicular structures [8, 10].

Presence of xanthoma cells may also cause diagnostic confusion with high-grade prostatic carcinoma. A panel of immunohistochemistry test including cytokeratin, PSA, prostatic acid phosphatase, leukocyte common antigen, and CD68 can be useful for differentiating between these two conditions by showing results more consistent with an inflammatory process [2, 6, 9].

Unlike prostatic adenocarcinoma, conservative treatment is the rule for xanthogranulomatous prostatitis given that, in many patients, the inflammation is self-limited and will resolve slowly over time. Surgical intervention may be needed if failure of conservative approach [4, 5, 7, 9, 11].

In exposed literature of xanthogranulomatous prostatitis surgery was usually performed because of severe lower urinary tract symptoms, or due to occurrence of complications that may require even prostatectomy as was reported by Wollin et al. [5, 6, 8–10].

Presentation of xanthogranulomatous prostatitis as prostatic abscess is rare, only reported three times in the English literature so far [6, 12, 13].

To the best of our knowledge we are reporting the fourth case revealed by prostatic abscess.

In contemporary practice, the incidence of prostatic abscess has significantly decreased due to the widespread use of antibiotics. Currently most prostatic abscess is seen in immunocompromised patients including diabetic and HIV patients.

Transrectal ultrasonography of the prostate represents the method of choice for diagnosis of prostatic abscess assessing location, size, and number of abscess cavities. It serves also as an important tool in treatment and follow-up. Computed tomography is mainly needed to accurately detect the extent of spread of the abscess, particularly to the ischiorectal fossa and perineum [12, 14].

Management of prostatic abscess is challenging especially in absence of guidelines for treatment.

Recently Abdelmoteleb et al. proposed an algorithm to manage this entity with Initial management entailing the use of broad spectrum antibiotics, and associated drainage is needed in cases of large abscess exceeding 1cm and in cases of failure to respond quickly to antibiotics with no signs of clinical improvement [14].

Drainage of prostatic abscesses can be achieved by different methods reported for their safety, feasibility, and effectivity: transrectal or transperineal ultrasound guided aspiration and endoscopic transurethral procedures or open drainage [12, 14, 15].

Currently, percutaneous transrectal or transperineal drainage appears to be the least invasive and safest treatment for drainage of prostatic abscesses. However, their major disadvantage is the risk of incomplete drainage of abscess especially in multifocal or multiloculated abscess.

Transurethral drainage of a prostatic abscess increases the likelihood of complete drainage avoiding repeated interventions and ensuring complete resection of the infected glandular prostate tissue [14, 15].

El-Shazly et al. reported favorable outcomes in patient treated with TUR which appears to be suitable for cases with multiple and diffuse prostatic abscesses or when aspiration does not show complete resolution of the fluid collection. They also described that their technique is different from formal TURP permitting eviction of complications associated with TURP especially retrograde ejaculation in relatively young patients [15].

Unlike conventional TURP, the technique of TUR (transurethral) drainage of prostatic abscess is not clearly defined and standardized including incision, deroofing, and resection.

In our case, transrectal ultrasound that could have provided us with an accurate assessment of the anatomical characteristics of the prostatic abscess (location, size, and the number of collections) was not done. Thus, TURP was performed in order to ensure a maximal excision of the abscessed prostatic tissue, minimizing the risk of incomplete drainage especially as the patient is diabetic.

## 4. Conclusions

In addition to the rarity of xanthogranulomatous prostatitis, occurrence of prostatic abscess on xanthogranulomatous prostatitis is even rarer.

Transurethral drainage appears to be the most efficient technique for management of prostatic abscess.

Accurate Imaging assessment of anatomical characteristics is mandatory in these patients to guarantee a complete drainage of abscess.

Final diagnosis of xanthogranulomatous prostatitis is exclusively based on histopathological examination.

Given clinical, biochemical, and imaging similarities with prostatic carcinoma and also paucity of data treating outcome of xanthogranulomatous prostatitis long-term follow-up is needed especially in patients with persisting elevated serum PSA values.
